# Treatment of *W*. *bancrofti* (Wb) in HIV/Wb Coinfections in South India

**DOI:** 10.1371/journal.pntd.0003622

**Published:** 2015-03-20

**Authors:** Kawsar R. Talaat, Subash Babu, Pradeep Menon, N. Kumarasamy, Jabin Sharma, Jeeva Arumugam, Kalaivani Dhakshinamurthy, Ramalingam Srinivasan, S. Poongulali, Wenjuan Gu, Michael P. Fay, Soumya Swaminathan, Thomas B. Nutman

**Affiliations:** 1 Laboratory of Parasitic Diseases, National Institute of Allergy and Infectious Disease, National Institutes of Health, Bethesda, Maryland, United States of America; 2 Department of International Health, Johns Hopkins Bloomberg School of Public Health, Baltimore, Maryland, United States of America; 3 National Institute for Research in Tuberculosis (formerly Tuberculosis Research Centre), Indian Council of Medical Research, Chennai, India; 4 YRG CARE, Chennai, India; 5 Division of Clinical Research, National Institute of Allergy and Infectious Diseases, Bethesda, Maryland, United States of America; Rosetta Genomics, ISRAEL

## Abstract

**Background:**

The disease course of human immunodeficiency virus (HIV) is often altered by existing or newly acquired coincident infections.

**Methodology/Principal Findings:**

To assess the influence of pre-existing *Wuchereria bancrofti* infection on HIV progression, we performed a case-controlled treatment study of HIV positive individuals with (FIL+) or without (FIL-) *W*. *bancrofti* infection. Twenty-eight HIV+/FIL+ and 51 matched HIV+/FIL- subjects were treated with a single dose of diethylcarbamazine and albendazole (DEC/Alb) and followed for a year at regular intervals. Sixteen of the HIV+/FIL+ subjects (54%) and 28 of the HIV+/FIL- controls (57%) were on antiretroviral therapy (ART) during the study. Following treatment, no differences were noted in clinical outcomes between the 2 groups. There also was no significant difference between the groups in the HIV viral load at 12 months as a percentage of baseline viral load (HIV+/FIL+ group had on average 0.97 times the response of the HIV+/FIL- group, 95% CI 0.88, 1.07) between the groups. Furthermore, there were no significant differences found in either the change in viral load at 1, 3, or 6 months or in the change in CD4 count at 3, 6, or 12 months between the 2 groups.

**Conclusions/Significance:**

We were unable to find a significant effect of *W*. *bancrofti* infection or its treatment on HIV clinical course or surrogate markers of HIV disease progression though we recognized that our study was limited by the smaller than predicted sample size and by the use of ART in half of the patients. Treatment of *W*. *bancrofti* coinfection in HIV positive subjects (as is usual in mass drug administration campaigns) did not represent an increased risk to the subjects, and should therefore be considered for PLWHA living in *W*. *bancrofti* endemic areas.

**Trial Registration:**

ClinicalTrials.gov NCT00344279

## Author Summary

In people living with HIV infection, simultaneous infections can adversely affect HIV disease. This has been seen with bacterial (tuberculosis), viral (cytomegalovirus), and parasitic infections (toxoplasmosis). Lymphatic filariasis is caused by a thin thread-like parasite that lives in the lymph vessels of infected people. It can cause significant disability. This infection is found in much of the same areas that high levels of HIV infection. We were interested in knowing if lymphatic filariasis changed the course of HIV infection in people with both diseases. In this study, the authors enrolled people in India who were living with HIV who either had or didn’t have filarial infection. All patients were treated for filariasis with 2 drugs, and then were followed for 1 year to see how their HIV disease progressed. No difference in HIV disease progression was found between the groups that did or did not have filariasis before treatment. The patients with HIV did well with the medicine for filariasis.

## Introduction

As the HIV epidemic continues in many parts of the world, more attention is being focused on strategies for prevention and management of HIV infection. In addition to highly active antiretroviral therapy (ART), the immune interactions between HIV and non-HIV co-infections have been examined. Several groups have examined the interaction of helminth infections with HIV, and many of these studies have been recently reviewed[1–3]. Some studies have shown that patients with HIV and concomitant helminth infections have higher viral loads which decrease upon anthelmintic treatment [4,5] whereas others have shown no effect of coincident helminth infections on viral load, CD4 count or HIV disease progression [6–9].

Few studies have looked at the interaction of filarial infections with HIV. In studies of patients with *Onchocerca volvulus* infection with and without HIV, those with HIV were found to have more significant skin disease [10] and were less likely to have antibodies to onchocercal antigens [11]. Despite these differences, HIV/*Onchocerca*-co-infected patients were as capable as HIV negative onchocerciasis patients of responding to treatment with ivermectin [12]. A cohort of HIV+ individuals coinfected with *Wuchereria bancrofti* (Wb) have been followed in Tanzania [13–15]. When treated with diethylcarbamazine (DEC), those who were circulating filarial antigen (CFA) positive (a surrogate for active infection) had a decrease in HIV viral load at 12 weeks whereas those who were CFA negative had an increase in viral load [14] following DEC administration. In a previous study, we have looked at the coinfection prevalence rates in India by surveying serum samples of HIV infected patients for filaria antigens. We found a prevalence of 5–9.5% of filarial antigenemia in HIV+ patients, similar to the prevalences found in the HIV negative population in the same region [16].

None of the studies to date has looked at the interactions between HIV and helminths outside of Africa. According to data from the Indian National AIDS Control Organization the 2011 estimated prevalence of HIV in adults in India was 0.27%. This results in about 2.1 million people infected with HIV [17]. The state of Tamil Nadu, in the south, has a higher than average adult HIV prevalence, with an estimated 133,000 infected adults [18]. Use of antiretrovirals is still rolling out in this population: as of January 2012, only 486,173 people were on ART [19].

## Methods

### Ethics statement

This study was approved by the Institutional Review Board of NIAID, the Ethics Review Boards at the Tuberculosis Research Centre (TRC, now the National Institute for Research on Tuberculosis) and YRGCare as well as by the Health Ministry Screening Committee of the Government of India. All subjects provided written informed consent.

### Study design

This was a prospective matched case-control study designed to compare HIV replication and progression of clinical disease between patients co-infected with HIV and Wb infection and patients that were HIV-positive but without Wb infection.

### Study medication

Albendazole was provided by Glaxo-Smith-Kline (GSK, Middlesex, UK) as part of the Global Program to Eliminate Lymphatic Filariasis (GPELF). Diethylcarbamazine (DEC) was from GSK-India, which is the same source that provided DEC for the GPELF. The study medication was stored at the two sites in Chennai where the study was conducted in the pharmacy under the control of a pharmacist or nurse. The study staff directly observed subjects as they took the medications.

### Study subjects

Existing or new adult subjects ≥18 years of age cared for at clinics run by YRGCare or the TRC at their sites in Chennai, India were recruited for this study. These subjects were all HIV+ by enzyme-linked immunosorbent assay (ELISA) and Western Blot testing. About half of those enrolled were on antiretroviral medication. Subjects were eligible if they were willing to participate and could give informed consent. Pregnant or lactating women were excluded, as were subjects that were acutely ill, had a hemoglobin <9 g/dl for women and <10 g/dl for men, had AST or ALT elevations >5 times the upper limit of normal, had evidence of acute HIV infection, or had active or known untreated tuberculosis.

Subjects willing to participate were screened for filarial antigenemia using a rapid immunochromatographic test (Filariasis Now, Binax, Portland, ME). Those found to be filarial antigen positive (cases) were enrolled, and data collected included demographics and clinical details. Each “case” who fulfilled the other inclusion criteria was matched for purposes of comparison with up to two HIV+, Wb-negative patients (controls) from the screened clinic cohort matched for age (within ±5 years), gender, viral load (matched to within ± 0.5 log HIV RNA copies), CD4 counts (matched to within the same range: <50; 50–100; 101–200; 201–300; >300) and for anti-viral regimes (that were similar enough in efficacy for purposes of effective comparison).

### Study procedures

At screening, all subjects had a thorough clinical assessment: a history and physical examination, blood for complete blood counts, liver functions (ALT, AST), CD4 count, serum storage for later analyses; stool samples were taken for examination of parasites. Female subjects underwent a urine pregnancy test. Potentially eligible subjects underwent a viral load. All enrolled subjects received a single dose of the combination of DEC (300 mg)/albendazole (400 mg). Subjects were followed at 1, 3, 6 and 12 months after receipt of the medication. At each visit, subjects were queried as to their interim history: any illnesses, new diagnoses, or changes in medications were noted. At all visits, a CBC and HIV viral load was scheduled; CD4 counts were scheduled at 3, 6 and 12 months. At the 12 month visit, all patients were retested for filarial antigenemia and, if positive, offered retreatment with DEC and albendazole.

Laboratory assays: Filarial antigen status was assessed in real time by immunochromatographic card test (Filariasis Now Binax, Portland, ME). Samples were stored, batched and grouped to run the *Wuchereria bancrofti* antigen ELISA (Tropbio Townsville, Australia) to quantitate levels of circulating filarial antigens. Filaria specific IgG and IgG4 levels were measured by ELISA as previously described [20].

### Study endpoints

The original endpoints of the study were the change in HIV viral load and the difference in clinical status between the cases and the controls one year after treatment with DEC and albendazole. We also examined the change in HIV viral load, CD4 counts and hemoglobin throughout the study.

### Statistical analysis

Differences in groups were tested by Wilcoxon-Mann-Whitney (WMW) test (for continuous responses), Fisher’s exact test (for categorical responses) and Wilcoxon signed rank (WSR) test for paired analyses. Additionally, a version of the WMW stratified by ART status was used [21].

Viral load was analyzed on a log_10_ scale. According to the guidelines for HIV treatment, baseline CD4 count was put into 1 of 4 categories: <200, 200–350, 350–500 and ≥500. Patients were classified as either on or not on ART during the study. Two patients started the study not on ART and began ART during the course of the study, and those two patients are treated as on ART for the main analyses. As a sensitivity analysis, all analyses were repeated with those two patients reclassified as off ART to see if there were substantial differences in the results.

A linear model was used to assess the association of the response log percentage of baseline viral load at 1 year with filarial co-infection, adjusting for other covariates (ART status, baseline hemoglobin, baseline CD4 count and baseline viral load) by including them in the model as main effects. We report filarial coinfection effects in terms of a multiplicative factor acting on the response (percentage of baseline viral load at 1 year) in the model. Similar linear models were done at 1 month, 3 months and 6 months, as well as with CD4 counts. All tests were 2-sided and at a significance level of 0.05. All analyses were conducted using the software R (version 2.15.2).

## Results

### Baseline

**Fig 1:**
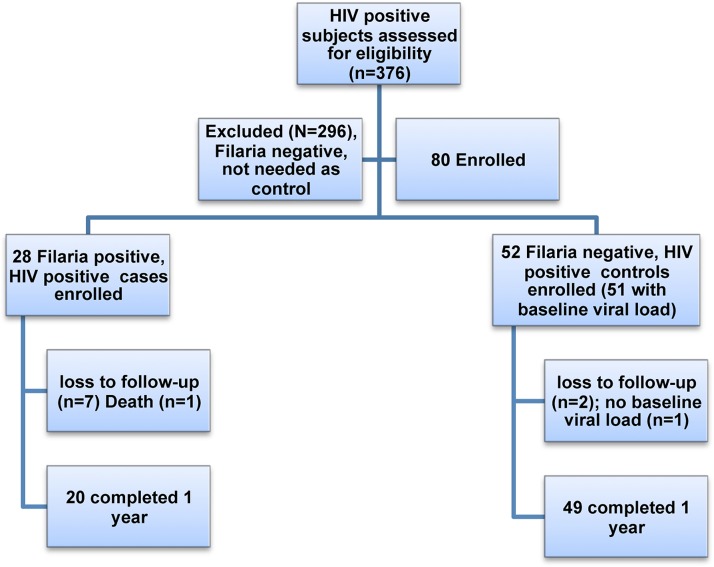
Consort diagram.

**Fig 2:**
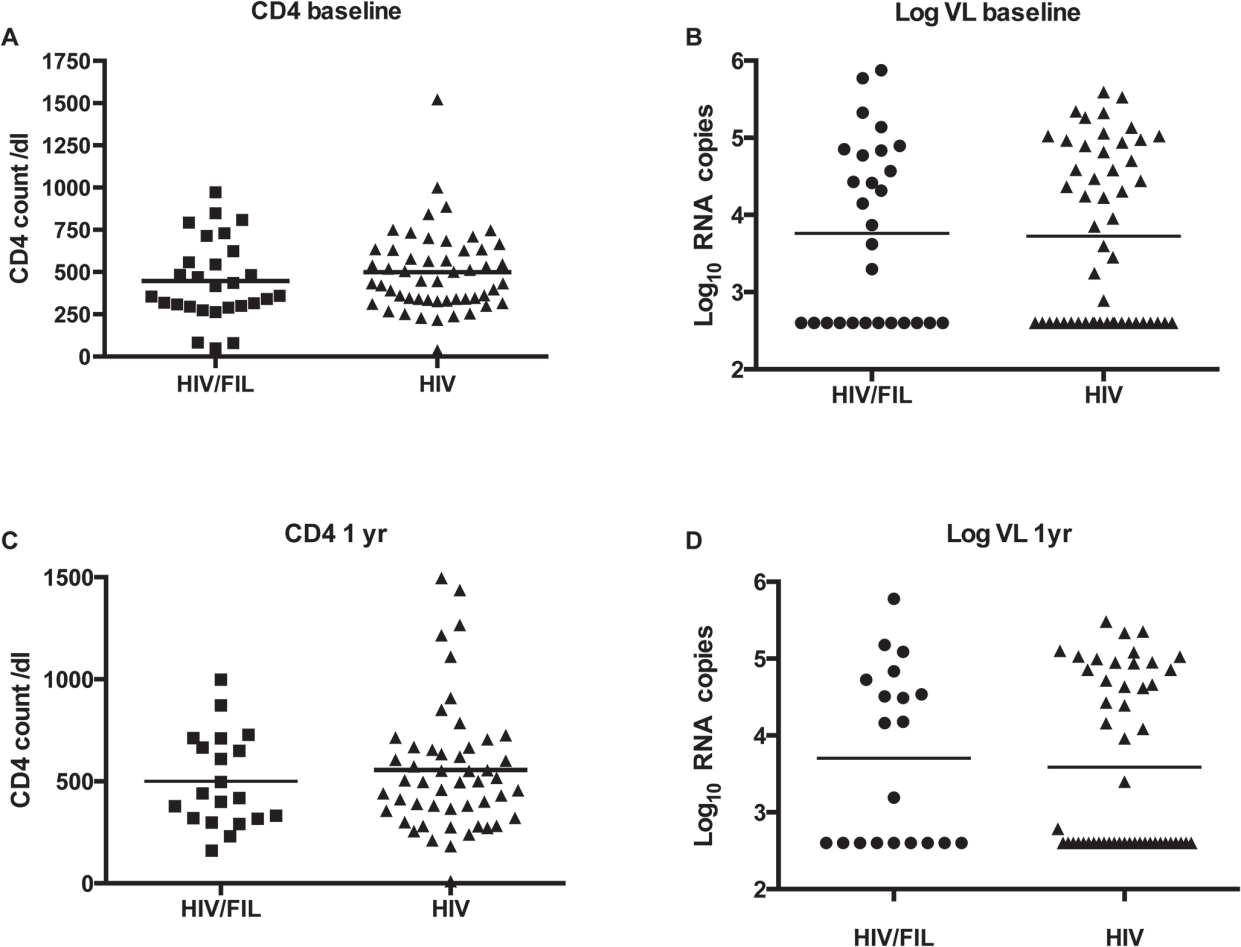
CD4 count and viral load comparisons at baseline and 1 year. Comparison of CD4 and viral load (VL) at baseline (**A** CD4 [count/dL]), **B** log VL [log_10_ RNA Copies]) and at 1 year (**C** CD4 count, **D** log VL) between HIV+/FIL+ cases and HIV+/FIL- controls.

376 subjects were screened (Fig. 1). Twenty-eight subjects were found to be filarial positive (HIV+/FIL+). These were matched with 52 HIV positive subjects who did not have Wb infection (filarial-negative) (HIV+/FIL-) as controls (one control subject had no baseline viral load). Baseline summary statistics are given in Table 1. Because the groups are matched, as expected there are no significant differences between the groups (Table 1, Fig. 2). Many subjects in both groups took co-trimoxazole and a multivitamin throughout the study. Less than 10% of subjects were positive for ova or parasites on stool exam, so this variable was not included in the analyses.

**Table 1:** Demographics.

	ENROLLED		
	HIV+/FIL+	HIV+/FIL-	Two-sided p-value
**Number**	**28**	52^	NA
**Gender**			
**N (%) female**	**12 (43%)**	23 (44%)	1.00 (Fisher’s exact)
**Language**			
**N (%) Tamil**	**19 (68%)**	38 (73%)	0.33 (Fisher’s exact)
**N (%) Telegu**	**9 (32%)**	11 (21%)	
**N (%) other/N/A**	**0 (0%)**	3 (6%)	
**Age**			
**GM (SD)**	**34.0 (6.9)**	34.8 (6.6)	0.5 (WMW)
**N (%) on ART**	**16 (57%)**	28 (54%)*	1.00 (Fisher’s exact)*
**Baseline Labs GM (95% CI)**			
**Hematology**			
**Hgb**	**12.7 (11.8, 13.5)**	13.1 (12.7, 13.4)	0.39 (WMW)
**WBC**	**6.4 (5.5, 7.4)**	6.2 (5.7, 6.8)	0.76 (WMW)
% **EOS**	**6.0 (4.3, 8.3)**	4.8 (3.9, 5.9)	0.23 (WMW)
**Plts**	**274 (220, 342)**	227 (208, 284)	0.18 (WMW)
**Liver function**			
**ALT**	**25 (21, 29)**	26 (22, 30)	0.99 (WMW)
**AST**	**26 (22, 29)**	28 (24, 31)	0.66 (WMW)
**HIV monitoring**			
**CD4 Count**	**370 (281, 488)**	443 (381, 515)	0.33 (WMW)
**VL (log**_**10**_)	**3.76 (3.32, 4.21)**	3.68 (3.37, 3.99)^	0.86 (WMW)
**Filaria antigen**			
**Circulating Ag**	**1123 (590, 2138)** ^**$**^	1.7 (1, 2.6)^$^	<0.0001 (WMW)

Abbreviations: HIV: Human Immunodeficiency Virus; FIL: Filaria (*W*. *bancrofti* antigen) positive; GM: geometric mean; SD: standard deviation; WMW: Wilcoxon-Mann-Whitney; ART: highly active antiretroviral therapy, N/A: Not available; %: percent. Hgb: Hemoglobin (gm/dL); WBC: White blood cell count (x 10^3^/mm^3^); EOS: eosinophils; Plts: platelet count (x10^3^/uL); ALT: alanine transaminase (SGPT, IU/L); AST: aspartate transaminase (SGOT, IU/L); VL: viral load; Ag: antigen.

^ 52 subjects were enrolled, but 1 subject did not have baseline HIV viral load value done. These statistics are on all 52 except for the viral load.

* In addition to the 28 subjects who got ART throughout, there were 2 (3.8%) subjects who got ART only part of the time during the study. Fisher’s exact test excluded those 2 subjects.

^$^ Measured on only n = 12 in the HIV+/FIL+ group and n = 39 in the HIV+/FIL- group.

### Clinical evaluation

The treatment with DEC/albendazole was well tolerated in our study subjects, with no adverse events attributed to the study medication. There were several unrelated significant illnesses in the study (1 HIV+/FIL+ subject developed malaria at 3.5 months, another HIV+/FIL- subject developed tuberculous meningitis at 4 months with leg weakness as a sequelae, and a third HIV+/FIL- subject had pulmonary tuberculosis and underwent a hysterectomy for an unrelated problem. One death occurred at 4.5 months in a HIV+/FIL+ subject who had been diagnosed and treated for pulmonary tuberculosis and cryptococcal meningitis prior to starting the study, and who had also been on ART for 1 year before enrollment. His death was attributed to a recurrence of the cryptococcal meningitis and possible tuberculoma. In addition to the death, 7 HIV+FIL+ subjects were lost to follow-up as were 2 HIV+/FIL- controls (Fig. 1), and additionally some subjects missed some viral load measurements at intermediate time points. No other subjects developed significant opportunistic infections while on treatment, although several had histories of tuberculosis or other infections prior to enrollment that had been treated.

### Laboratory evaluation

**Fig 3:**
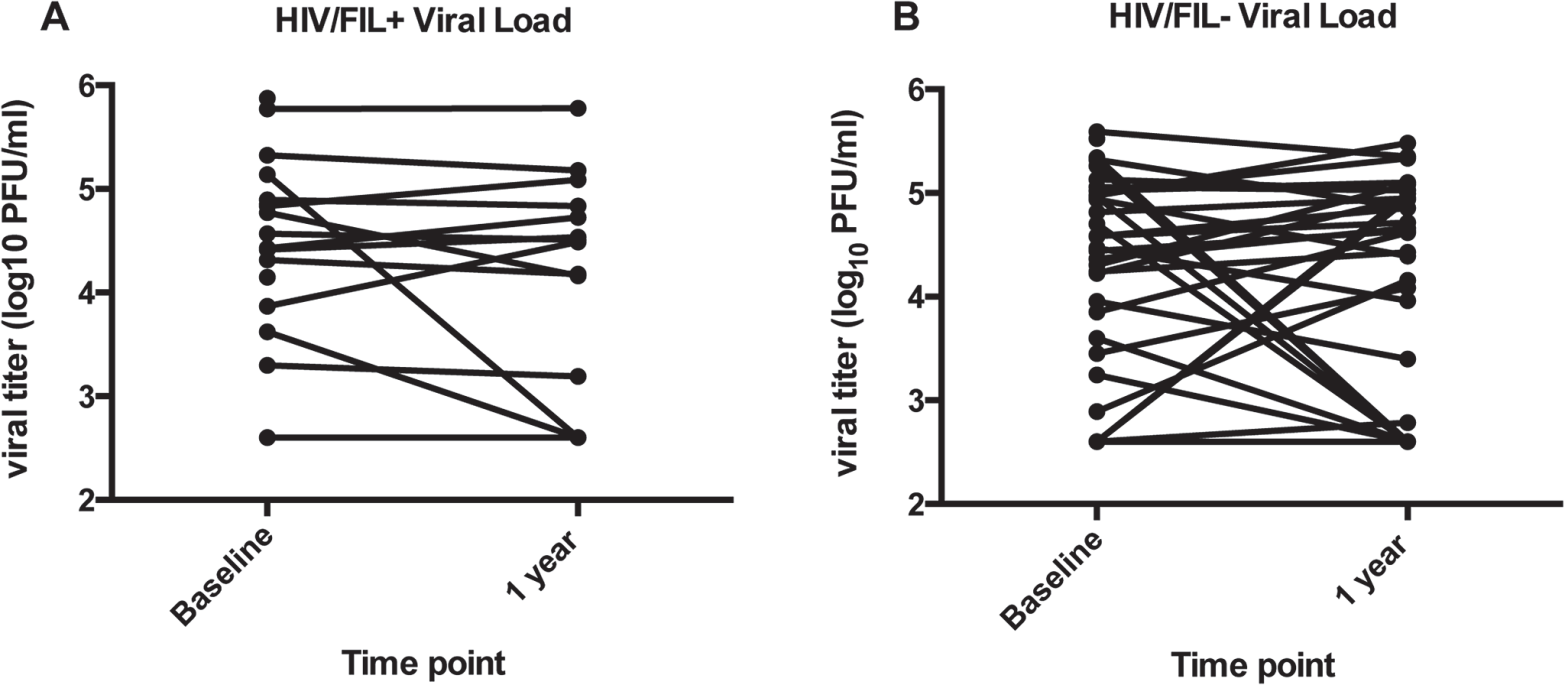
HIV viral load by over time by individual. HIV VL at baseline and 1 year in HIV+ /FIL+ individuals (**A**) and HIV+/FIL- individuals (**B**).

Following DEC/Alb, subjects were followed at 1, 3, 6, and 12 months following drug administration. At one year, the planned primary endpoint, we have VL measurements on 20 (HIV+/FIL+) and 49 (HIV+/FIL-) subjects. One of the HIV+/FIL+ died. There was no significant difference between the two groups (HIV+/FIL+ and HIV+/FIL-) in the percent of baseline viral load at one year (counting the subject that died as ranked highest for response, and the 2 subjects that got ART for part of the time as having ART) by stratified WMW test, stratified by ART (p = 0.41) (Fig. 2, Fig. 3). To estimate an effect of HIV+/FIL+, we ran a linear model on the log percent change. We find that, controlling for ART, on average those with HIV+/FIL+ have about 0.97 times the percent change of viral load at one year than those with HIV+/FIL- (95% CI 0.88, 1.07). Repeating the analysis at different time points post baseline, we get similar results. We get similar results in all these models if we additionally control for baseline hemoglobin, baseline CD4 count and baseline viral load, or if the 2 subjects who started ART after baseline are treated as not on ART.

When we examined the subset of subjects (n = 27) who were not on antiretrovirals (9 HIV+/FIL+ subjects, 18 HIV+/FIL- subjects), we found that at 1 month following DEC/Alb, there was a significant difference in the percent change of the HIV log_10_ viral load between the 2 groups with the HIV+/FIL+ subjects showing a GM increase of 5.1% [95%CI −3.7 to 13.6%] compared to the HIV+/FIL- subjects who showed a change of −2.2% [95% CI −6.3 to 2.0%]; p = 0.05 WMW. This difference in the changes in viral loads was not sustained throughout the remaining 3, 6 or 12 month time-points (at 12 months, the HIV+/FIL+ subjects not on antiretrovirals [n = 9] had a mean change of −1.0 logs [95% CI −5.2 to 3.2], and the HIV+/FIL- subjects (n = 22) had a mean change of 2.6 logs [95% CI −7.9 to 13.1] p = 0.2).

**Fig 4:**
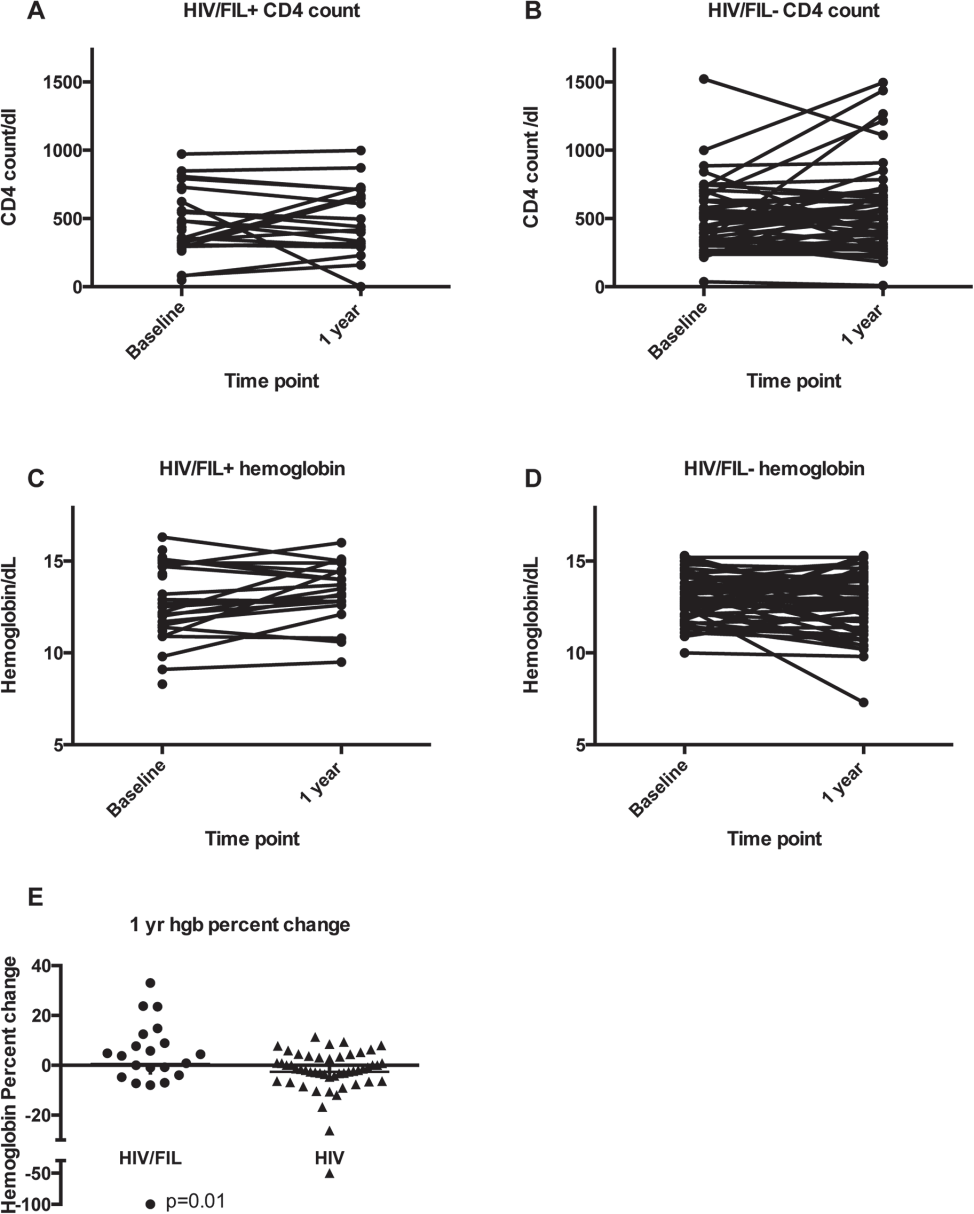
CD4 counts and hemoglobin levels over time. CD4 counts (**A** and **B**) and Hemoglobin levels (Hgb) (**C** and **D**) at baseline and 1 year between HIV+ /FIL+ individuals (**A**, **C**) and HIV+/FIL- individuals (**B**, **D**). **E**. Hemoglobin percent change at 1 year between HIV+/FIL+ and HIV+/FIL- individuals.

CD4 counts were assessed at 3, 6 and 12 months. Analogous to the analysis with viral load, we use the linear model with log percent change in CD4 count, and express the effects as how many times larger the average percent change is for the HIV+/FIL+ group than for the HIV+/FIL- group. We find that there are no significant differences between the groups: at 1 year (1.10; 95% CI 0.88, 1.38; p = 0.40) (Figs. 2 and 4), 3 months (0.99; 95% CI 0.82, 1.20; p = 0.92), and 6 months (1.05; 95% CI 0.89, 1.24; p = 0.55).

Hemoglobin was assessed at 1, 3, 6 and 12 months. Again we use the linear model with log percent change in hemoglobin, and express the effects as how many times larger the average percent change is for the HIV+/FIL+ group than for the HIV+/FIL- group. We find that there are significant differences between the groups at 1 year (1.08; 95% CI 1.02, 1.15; p = 0.01) (Fig. 4 C-E). We do not see those significant effects at other times: 1 month (1.00; 95% CI 0.96, 1.03; p = 0.78), 3 months (1.01; 95% CI 0.95, 1.07; p = 0.78), and 6 months (1.04; 95% CI 0.99, 1.08; p = 0.11).

**Fig 5:**
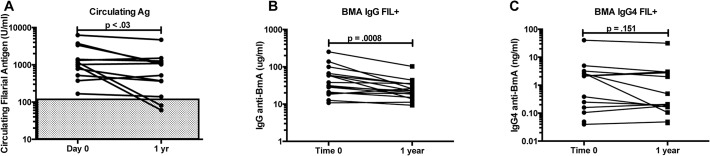
A. *Wuchereria bancrofti* antigen and antibody levels over time. Circulating *Wuchereria bancrofti* antigen levels in HIV+/FIL+ individuals at baseline and 1 year. **B**. BMA-specific IgG in HIV+/FIL+ subjects at baseline and 1 year. **C**. Total IgG4 levels in HIV+/FIL+ subjects at baseline and 1 year.

To examine the efficacy of single dose DEC/Alb in HIV+/FIL+ patients, quantitation of circulating filarial antigen (CFA) levels was assessed at baseline and at 1 year in those for whom samples were available (n = 12). The GM CFA level in those who were positive at baseline was 1123 IU/ml (95% CI 590–2138); at 1 year, that level had decreased to 534 IU/ml (95% CI 235–1215) (p = 0.03 WSR) (Fig. 5). Only 2 subjects who were CFA positive at the start of the study became negative after 1 year. No CFA negative subjects became positive at the end of the study. Treated filaria positive subjects also saw a decrease in the GM filaria-specific IgG and IgG4. IgG decreased by 43% (from 40.4 μg/ml (CI 25–64) at baseline to 23.7 μg/ml (CI 17–32) at 1 year (p = 0.0008) and IgG4 decreased by 31%, although not statistically significant (p = 0.1514): from 0.65 ng/ml (CI 0.19–2.2) baseline to 0.52 ng/ml (CI 0.16–1.7) at one year (Fig. 5).

## Discussion

In this study, we attempted to look at the progression of HIV disease in patients with *W*. *bancrofti*/HIV co-infection compared with those with HIV alone after a one-time treatment with DEC/Alb, the standard therapy used worldwide (except in Africa) for mass drug administration programs. In addition, we also looked at biomarkers as surrogates for disease progression (CD4 count, viral load). We found no difference in the clinical outcomes of the subjects one year after treatment. We did find a transient and significant increase in viral loads in those Wb/HIV co-infected subjects not on ART after 1 month, though this difference in VLs between the Wb-infected and -uninfected HIV+ subjects equalized for the remainder of the study. When we examined CD4 counts at 3, 6 and 12 months, or viral loads and hemoglobin values at 1, 3, 6, and 12 months we found no difference between the 2 groups with the exception of higher hemoglobin values in the co-infected group at 1 year. The protective effect of filarial infection on infection-related anemia is lent support by a study of filarial/malaria co-infection in Africa [22].

In the HIV+/FIL+ subjects, circulating filaria antigen levels and filaria-specific IgG and IgG4 were lower in at 1 year than at baseline (although this did not achieve significance for the IgG4), suggesting that the treatment was effective in decreasing filarial antigen load.

The present study has several limitations, the most significant being the small sample size. We found a much lower than expected rate of *W*. *bancrofti* positivity in the HIV population in and around Chennai, although our sample size calculations were based on our 2004 prevalence data in which we found that 9.5% of HIV positive subjects were filarial also antigen positive [16]. India, like other LF endemic countries of the world, is participating in the Global Program to Eliminate Lymphatic Filariasis (GPELF), and is distributing single dose diethylcarbamazine (DEC) and albendazole on a population-wide level in some endemic regions. In the period leading up to and during the study, there was a large drop in the prevalence of *W*. *bancrofti* infection in the population with little transmission occurring in the urban areas from which the patients were recruited. It is possible that the MDA programs have lowered the prevalence and incidence of lymphatic filariasis infection to levels much lower than we had seen previously.

At the time the study was planned, India was just starting to use ART. By the time we had enrolled all of our subjects and controls, >50% of them were on ARTs. While this was a distinct benefit to our subjects, it did make it difficult for us to see a difference in viral loads between subjects and controls since many of them were already optimally virally suppressed at baseline.

Finding a difference in the course of HIV through treatment of a concomitant helminth infection is quite difficult [13,15]. However, in one randomized blinded placebo controlled crossover study in Tanzania of 34 HIV+ individuals, there was a significant salutary effect on VL at 12 week in filarial-infected individuals at 12 weeks [14]. ARVs were not used, a potential difference between this study and the present study.

Other groups have looked at the interactions with HIV and other non-filarial helminth infections with mixed findings [4,5,10,11,23–28]. Two studies have shown that anthelmintics decreased HIV viral load in helminth-infected HIV+ adults [4,14] whereas others have shown just the opposite [5]. In most other studies there has been demonstrated little to no difference in viral load after anthelmintic treatment [6–8].

The effects of helminth coinfection on CD4 counts in HIV-coinfected subjects are also conflicting. In several studies in Africa, the presence of helminth infection has been shown to adversely affect CD4 counts [26,28,29], although other studies have shown either a protective effect by helminths on CD4 counts [24,30] or no effect whatsoever [7,24,31]. A study from Uganda showed no overall effect of empiric deworming in HIV positive patients, however female patients who received deworming had a greater increase in CD4 count at 1 year that was not sustained at 2–3 years. In addition, women had an increase in hemoglobin after deworming that was not seen in men [9]. Studies of HIV and filarial co-infection in the future will be increasingly difficult as the prevalence of filarial infections decrease and more patients with HIV infection receive ART. Future studies trying to examine this issue will be limited to areas of high prevalence of both infections or as sub-studies within large cohorts in order to achieve a sample size that can address some of the remaining questions.

In conclusion, our study has clearly demonstrated that *W*. *bancrofti* infection, the most prevalent filarial infection of humans, failed to influence HIV-related clinical status, VL or CD4 counts at baseline and following treatment with DEC/ALB though there was an apparent filarial-induced increase in VL after DEC/ALB in ART-naive coinfected individuals. Our data suggest that like with vaccinations in HIV-infected individuals that the benefits of MDA in *W*. *bancrofti* and other related infections far outweigh a miniscule transient risk in parasite/HIV coinfections.
